# Intraosseous Schwannoma of the Calcaneus: A Rare Tumor of the Bone

**DOI:** 10.1155/2018/9824025

**Published:** 2018-10-02

**Authors:** Bahtiyar Haberal, Duygu Turkbey Simsek, Ekin Kaya Simsek

**Affiliations:** ^1^Department of Orthopaedics and Traumatology, Baskent University Hospital, Ankara, Turkey; ^2^Department of Pathology, Baskent University Hospital, Ankara, Turkey

## Abstract

Schwannomas (also called neurilemmomas) are slow-growing nerve sheath tumors derived from Schwann cells. However, intraosseous schwannoma is a rare entity with an incidence of only 0.2% in overall primary bone tumors. The purpose of this case report is to present a case of an intraosseous schwannoma of the calcaneus. A 35-year-old woman was admitted to our outpatients' clinics with a complaint of long-time right heel pain (for approximately eight months). After a suspicious cystic lesion was observed on the patient's plain radiograph examination, a magnetic resonance imaging (MRI) was performed. The MRI showed a 22 × 20 mm intraosseous cystic lesion at the posterior part of the calcaneus. Extended curettage and iliac bone grafting were performed. In the presence of postoperative histopathologic and immunohistochemical examination, histopathologic diagnosis of the patient was reported as intraosseous schwannoma. After 4 weeks of nonweight-bearing, she completely recovered with no pain. In conclusion, intraosseous schwannoma of the calcaneus must be kept in mind for patients who have chronic heel pain.

## 1. Introduction

Schwannomas (also called neurilemmomas) are slow-growing nerve sheath tumors derived from Schwann cells. However, intraosseous schwannoma is a rare entity with an incidence of only 0.2% in overall primary bone tumors [1]. In the literature, the most commonly affected bone is the mandible. Other commonly affected bones are long bones (tibia, femur, fibula, humerus, radius, and ulna), vertebrae, and the other bones including patella, petrous apex, scapula, and metacarpals. The tumor can affect the bone with three possible mechanisms: tumors may occur directly within the interior of the bone, tumors may originate from the nutrient canal, or it may be extraosseous that is destructive for the bone [2]. Intraosseous schwannoma may also be associated with neurofibromatosis type 1 (von Recklinghausen's disease) and Carney syndrome [3, 4]. However, only four cases of intraosseous schwannoma of the calcaneus have been reported in the English literature [5–8]. The purpose of this case report is to present a case of an intraosseous schwannoma of the calcaneus.

## 2. Case Presentation

A 35-year-old female was admitted to our outpatients' clinic with a compliant of an eight-month history of right heel pain which had increased gradually in the past two months. The pain of the patient often occurred at night and it was not related with daily or sporting activities and most commonly felt at rest. Her symptoms started insidiously and she did not mention any trauma. She had been smoking 20 cigarettes per day for 10 years, and she did not suffer from any medical condition. Furthermore, no hereditary disease was found in the patient's family history.

**Figure 1:**
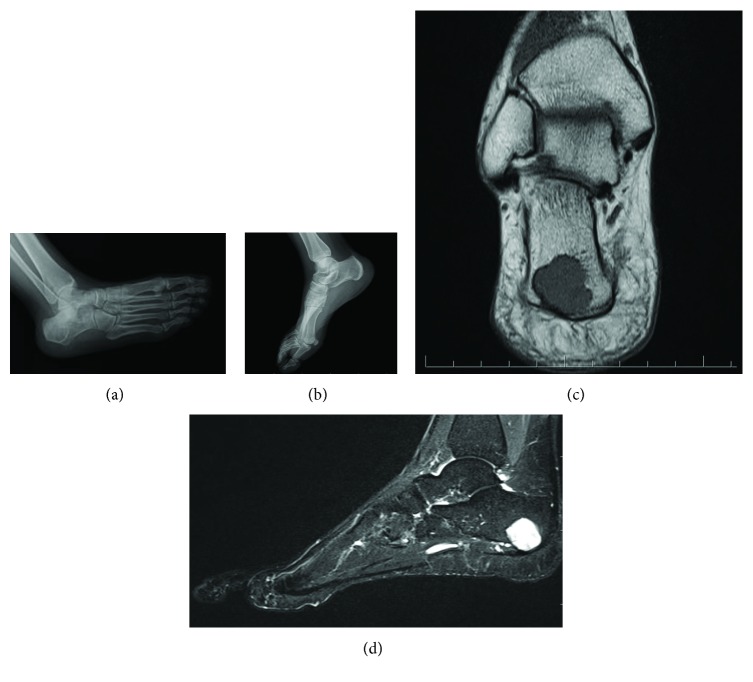
(a, b) Oblique and lateral plain radiograph of the patient's foot shows lytic lesion with sclerotic rims in the long axis of calcaneus. (c) Coronal T1-weighted MRI image shows hypointense signaling, cortical involvement, and well-defined borders. (d) Sagittal T2-weighted MRI image shows hyperintense signaling and heterogenous appearance.

Physical examination revealed no abnormal findings. Her blood tests were all normal, including all the inflammatory markers. The patient underwent plain radiograph examination and a 21-millimeter-diameter cystic lesion was observed in the long axis of the calcaneus. The magnetic resonance imaging (MRI) examination was applied for the confirmation and it showed 22 × 20 mm intraosseous cystic lesion at the posterior part of the calcaneus which revealed hypointense signal on T_1_-weighted images and hyperintense signal on T_2_-weighted images (Figure 1). The contrast-enhanced MRI was not available for this patient.

Although the lesion was radiologically benign, extended curettage and iliac bone grafting were planned to exclude the malignancy risk that may be caused by smoking history. The operation was performed under spinal anesthesia. After bone grafting from the right ilium was completed, tourniquet was applied to the right thigh. A lateral approach to the calcaneus was used. After opening a 10 × 10 mm valve from the lateral aspect of the calcaneus, the cyst was excised and the cyst walls were debrided by burr. After preparation of the area, the cavity was filled with bone grafts.

**Figure 2:**
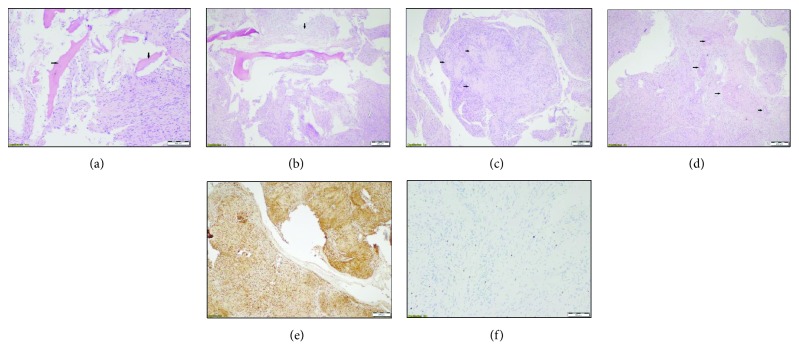
(a) Histological examination reveals bundles of elongated cells with spindle-shaped nuclei between mature bone fragments (black arrows) (H&E ×10). (b) The hallmark of a schwannoma is the pattern of alternating Antoni A (black arrow) and B (white arrow) areas (H&E ×4). (c) Verocay bodies (black arrow), formed by two compact rows of well-aligned nuclei separated by fibrillary cell processes (H&E ×4). (d) Thickened hyalinized vessels (black arrow) are irregularly scattered in the lesion which are characteristic of schwannomas (H&E ×4). (e) Cytoplasmic and nuclear immunostaining of neoplastic cells with S-100 protein (magnification ×10). (f) Ki-67 proliferation index was observed around 5% at the highest level (magnification ×10).

Microscopic examination revealed compact hypercellular areas with spindle cells which show nuclear palisading around fibrillary process in some areas. There was no mitotic activity. Diffuse expression of S-100 protein was observed with immunohistochemical staining, and Ki-67 proliferation index was observed around 5% at the highest level (Figure 2).

**Figure 3:**
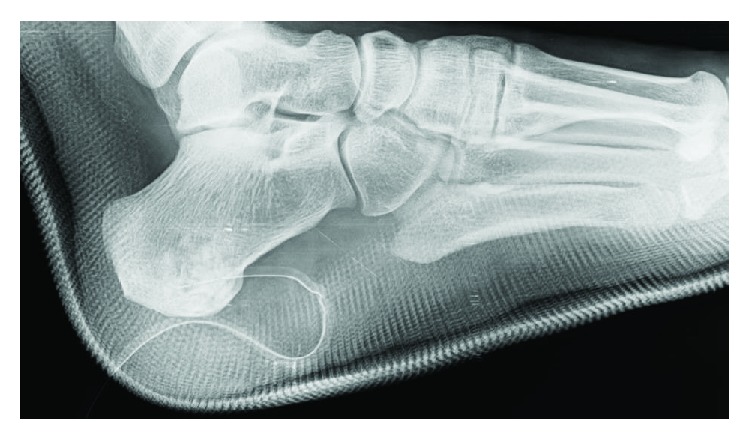
Postoperative lateral plain radiograph of the patient's foot shows radiopacity of the bone grafts at the surgery site.

In the presence of these findings, histopathologic diagnosis of the patient was reported as intraosseous schwannoma. Postoperatively, short leg splint was applied until the sutures were removed (Figure 3). Ankle function exercises were begun at the second week after surgery. After 4 weeks of nonweight-bearing, she completely recovered with no pain at the end of the 2nd month.

## 3. Discussion

Schwannomas are benign peripheral nerve sheet tumors that originate from the Schwann cells. Because the sensory nerves contain more Schwann cells than other nerves in their axons, they developed schwannomas more frequently. Schwannomas are common in soft tissues of the head and neck, and very few schwannoma are associated with bones. The mandible and sacrum are frequent sites because the mandibular nerve is a predominant sensory nerve and many sensorimotor nerve roots pass through the sacral foramina. Primary schwannomas of the bone are slow-growing lesions which arise from the nerves that innervate the bone and often centering on the medullary cavity and mimicking other primary bone tumors [4].

In the literature, the most common symptom was nonspecific, slow-onset pain and periodic swelling or it may be asymptomatic. In some series, it had been reported that impairment of sensory and motor functions and pathological fractures may be seen. Schwannomas are usually presenting in the second to fifth decades of life, and they are rare in children [9, 10]. Additionally, female patients are slightly more often affected than male patients [4].

The radiological findings of the intraosseous schwannomas are nonspecific, and it is not helpful for the differential diagnosis but they are always suggesting a benign nature. Plain radiographic features of intraosseous schwannoma included a well-defined lytic lesion with thin sclerotic rims, lobulated or trabeculated contours, cortical expansion and erosion, and absence of internal calcification [2]. Our case showed a lytic lesion with sclerotic rims, and it is difficult to differentiate from other benign bone lesions such as bone cyst, aneurysmal bone cyst, benign chondroblastomas, giant cell tumors, fibrous dysplasia, and nonossifying fibroma; thus, MRI examination was performed. On MRI, schwannomas are solid lesions and they tend to be isointense to skeletal muscle on T_1_-weighted imaging and hyperintense and heterogeneous on T_2_-weighted images [10]. MRI scans in our case showed that the lesion was expansive with cortical involvement and it has heterogenous appearance with well-defined borders.

Microscopic evaluation is the most important step in establishing a definitive diagnosis, and histological features are similar to soft tissue Schwannomas. Schwannomas of the soft tissue contain two components: a highly ordered cellular component (Antoni A) and a loose myxoid component (Antoni B) [9]. The main difference between interosseous and soft tissue schwannomas is the presence of a higher degree of cellularity with subtle palisading and poorly formed Verocay bodies in the former [11]. The histology of our patient was consistent with that of a soft tissue schwannoma, consisting mainly Antoni A and Antoni B, and immunohistochemical study showed that the lesion was positive for S-100 protein. The S-100 protein is important for histological differential diagnosis of the desmoplastic fibroma, well-differentiated fibrosarcoma, fibrous dysplasia, and nonossifying fibroma.

Intraosseous schwannoma is associated with a good prognosis, and malignant transformation has not been reported. Therefore, the most recommended treatment is curettage and bone grafting [12]. In the case series, recurrence rate was high (16%) with incomplete tumor excision while recurrence was not observed in complete surgical removal [13]. In this patient, we performed extended curettage and bone grafting in accordance with the literature.

In conclusion, because an intraosseous schwannoma of the calcaneus is extremely rare, it may be difficult to differentiate intraosseous schwannoma from other painful, radiographically benign-looking lytic bone lesions of the calcaneus. Nevertheless, it must be kept in mind for patients who have chronic, insidious heel pain.
